# Consumption of Sodium and Its Ratio to Potassium in Relation to All-Cause, Cause-Specific, and Premature Noncommunicable Disease Mortality in Middle-Aged Japanese Adults: A Prospective Cohort Study

**DOI:** 10.1016/j.tjnut.2024.12.020

**Published:** 2024-12-27

**Authors:** Ribeka Takachi, Marina Yamagishi, Atsushi Goto, Manami Inoue, Taiki Yamaji, Motoki Iwasaki, Kazumasa Yamagishi, Hiroyasu Iso, Shoichiro Tsugane, Norie Sawada

**Affiliations:** 1 Department of Food Science and Nutrition, Nara Women’s University Graduate School of Humanities and Sciences, Kitauoya-higashimachi Nara, Japan; 2 Department of Health Data Science, Graduate School of Data Science, Yokohama City University, Yokohama, Japan; 3 Division of Cohort Research, National Cancer Center Institute for Cancer Control, Chuo-ku, Tokyo, Japan; 4 Division of Prevention, National Cancer Center Institute for Cancer Control, Chuo-ku, Tokyo, Japan; 5 Division of Epidemiology, National Cancer Center Institute for Cancer Control, Chuo-ku, Tokyo, Japan; 6 Department of Public Health Medicine, Institute of Medicine, and Health Services Research and Development Centre, University of Tsukuba, Tsukuba, Ibaraki, Japan; 7 Department of Public Health, Graduate School of Medicine, Juntendo University, Bunkyo-ku, Tokyo, Japan; 8 National Center for Global Health and Medicine, Shinjuku-ku, Tokyo, Japan; 9 International University of Health and Welfare Graduate School of Public Health, Minato-ku, Tokyo, Japan

**Keywords:** sodium, sodium-to-potassium ratio, premature death, NCD, mortality

## Abstract

**Background:**

Reducing premature noncommunicable disease (NCD) mortality is a global challenge. Sodium is thought to increase risk of NCDs via an effect of salt per se or high-salt foods on hypertension-induced cardiovascular disease (CVD) and gastrointestinal cancer. Further, relative risk of CVD is reportedly more closely associated with sodium-to-potassium ratio than that with sodium alone. However, few studies have investigated the effect of consumption of sodium or its ratio to consumption of potassium on risk of premature NCD death.

**Objectives:**

We examined associations between intake of sodium and sodium-to-potassium ratio and risk of all-cause and cause-specific death, including premature NCD, in a Japanese prospective cohort study.

**Methods:**

During 1995–1998, a validated food frequency questionnaire was administered in 11 areas to 83,048 men and women aged 45–74 y. During 1,587,901 person-years of follow-up until the end of 2018, 17,727 all-cause deaths and 3555 premature NCD deaths were identified.

**Results:**

Higher sodium intake was significantly associated with increased risk of all-cause and premature NCD mortality, but not all NCD mortality, among men: multivariate hazards ratios for the highest compared with lowest quintiles (HR) were 1.11 (95% CI: 1.03, 1.20; *P*-trend < 0.01) for all-cause and 1.25 (95% CI: 1.06, 1.47; *P*-trend < 0.01) for premature NCD mortality. When intakes were expressed as ratio to potassium intake, these associations (HR of all-cause: 1.19, 95% CI: 1.11-1.27; *P*-trend < 0.01; HR of premature NCD: 1.27, 95% CI: 1.10, 1.46; *P*-trend < 0.01), including associations with cancers (HR: 1.18, 95% CI: 1.07, 1.31; *P*-trend = 0.02), were strengthened in men.

**Conclusions:**

This prospective cohort study showed that both sodium intake and sodium-to-potassium ratio are associated with increased risk of all-cause and early NCD mortality in middle-aged men.

## Introduction

High sodium intake is reported to be a major contributor to noncommunicable disease (NCD) mortality worldwide [1], especially for cardiovascular diseases (CVD). Efforts have been made around the world to reduce salt intake [[2], [3], [4]]; nevertheless, salt remains a major risk factor for NCDs among dietary factors [5]. A review reported a U-shaped association between sodium intake and mortality, indicating that the beneficial effect of sodium reduction on blood pressure outweighs the harmful effect on hormones and lipids (such as plasma renin and aldosterone) at relatively higher levels of sodium intake [6]. Asian people, including Japanese, have the highest levels of salt intake in the world [1,7].

The mechanism most frequently proposed to explain the association between sodium and CVD involves cerebrovascular disease–related mortality via hypertension. The effect of hypertension on increased risk of cerebrovascular disease–related mortality appears stronger in middle-aged to early-aged individuals than that in later-aged individuals [8]. Accordingly, the magnitude of the impact of sodium intake on mortality in middle-aged vis-a-vis early-aged people should also be considered. Of note, Sustainable Development Goals Target 3.4 from the WHO aims to reduce premature mortality from NCD by one-third through prevention and treatment [9]. Further, the WHO defines NCDs as composite indicators of CVD (including coronary artery disease and cerebrovascular disease), cancers, chronic respiratory diseases, and diabetes (including kidney disease deaths caused by diabetes). To our knowledge, however, no prospective study has addressed the association between diet, including sodium intake or sodium-to-potassium ratio, and premature NCD mortality itself as outcomes.

Importantly, any investigation of sodium and CVD should include sodium-to-potassium ratio, on the basis that when examined as a ratio to potassium, - of which vegetables are a major source, and effective themselves against CVD [[10], [11]] - the associations are reportedly stronger than when sodium intake is evaluated alone [12,13]. In this study, we examined associations between the intake of sodium and the sodium-to-potassium ratio and risk of premature NCD mortality in a population-based prospective cohort study in Japan.

## Methods

### Study population

The Japan Public Health Center-based Prospective (JPHC) study was established in 1990 (cohort I) and 1993 (cohort II). The study population of 140,420 men and women included all registered Japanese inhabitants of 11 public health center areas identified by population registries in the local municipalities. By age, participants were 40–59 y in cohort I and 40–69 y in cohort II. The study design has been described in detail elsewhere [14]. The study protocol was approved by the institutional review board of the National Cancer Center, Tokyo, Japan.

Information based on a self-reported questionnaire survey was obtained at the initial survey (first) and at the 5-y (second) and 10-y (third) follow-ups. Because information on food intake frequency was more comprehensive in the second survey (1995 for cohort I and 1998 for cohort II) than the first, dietary exposure for this study used the second survey as starting point. The survey questionnaires also enquired about medical history and lifestyle factors, including smoking and alcohol consumption.

**FIGURE 1:**
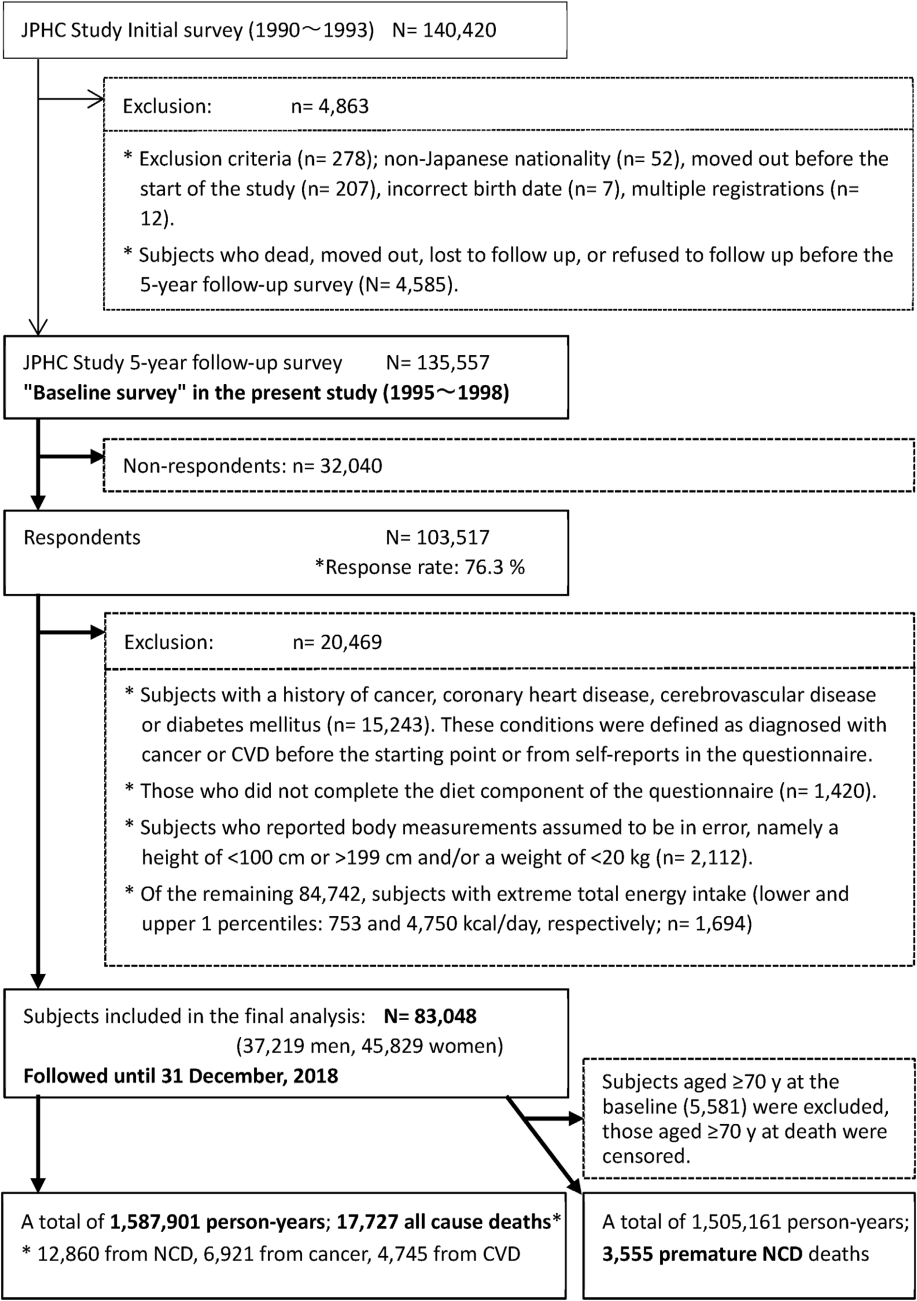
Flowchart for definition of subjects for this study. CVD, cardiovascular disease; JPHC, Japan Public Health Center-based Prospective; NCD, noncommunicable disease.

After exclusion of 4863 persons with non-Japanese nationality, declined participation, had died, moved out of the study area, or were lost to follow-up before the starting point (5 y after initiation), 135,557 participants remained eligible for participation. Of these, 103,517 participants responded (response rate: 76.3%) and were included in this study (flow of participant definitions is shown in Figure 1). Further, of the 103,517 respondents, we excluded participants with a history of cancer, coronary artery disease, cerebrovascular disease, or diabetes mellitus (*n* = 15,243) and those who did not complete the diet component of the questionnaire (*n* = 1420). Participants with a history of these conditions were defined as diagnosed with cancer or CVD before the start point or from self-reports in the questionnaire. Moreover, we also excluded 2112 who reported body measurements assumed to be in error, namely a height of <100 cm or >199 cm and/or a weight of <20 kg. Of the 84,742 participants, we also excluded 1694 with extreme total energy intake (lower and upper 1 percentiles, namely 753 and 4750 kcal/d, respectively), leaving 83,048 participants (37,219 men, 45,829 women) for the final analysis. Respondents excluded due to medical history or concerns about incomplete responses were ∼5 y older than those included in the analysis for both sexes, with a mean (SD) age of 59.8 (8.0) and 60.5 (8.2) y for 11,121 men and 9348 women, respectively.

### Food frequency questionnaire

The food frequency questionnaire (FFQ) enquired about usual consumption of 138 foods and beverages, including 4 seasonings (ketchup, dressing, mayonnaise, and Worcester sauce), during the preceding year in standard portion/unit sizes, specified by food item in the amount choices of small (50% smaller), medium (same as standard), and large (50% larger) and in 9 categories of frequency [15,16]. Food items and supplemental questions listed in the FFQ were as reported previously [16]. Energy and nutrient intake, including sodium, were calculated based on the Standardized Tables of Food Composition, 7th revised edition [17]. Calculation of sodium intake in the FFQ is detailed elsewhere [18]. In brief, cooking salt and soy sauce intake was estimated using the responses for typical cooking methods for individual food groups, as well as table salt and soy sauce added to these food groups. Sodium intake from miso soup was also considered and further adjusted by taste preference.

The validity of the FFQ in assessing sodium and potassium intake has been confirmed [15,19,20]. Respective Spearman correlation coefficients (CCs) of energy-adjusted sodium intake derived from the FFQ compared with that derived from 28-d (14-d in 1 area) dietary records in subsamples (*n* = 215 in cohort I, 350 in cohort II) of men and women were 0.47 and 0.50 in cohort I and 0.32 and 0.31 in cohort II, respectively. Corresponding respective CCs for potassium intake were 0.49 and 0.40 in cohort I and 0.48 and 0.50 in cohort II, respectively. Moreover, on comparison with two 24-h urinary excretions measured at an interval of 7 mo, CCs of men and women respectively were 0.42 and 0.30 for sodium and 0.40 and 0.24 for potassium, respectively. Respective CCs for the reproducibility of intakes of sodium and potassium derived from the FFQs given 1 y apart were 0.49–0.67 and 0.45–0.63, respectively [21,22].

### Follow-up

Participants were followed up from the start point until 31 December, 2009, for participants living in Tokyo (*n* = 2824); 31 December, 2012 for those living in Osaka (*n* = 8102); and 31 December, 2018 for all others. Where residence status changed, including survival, data were obtained annually from the residential registry of the local area or through the municipal office in their new area for those who moved. Mortality data for participants in the residential registry are transferred to the Ministry of Health, Labour, and Welfare and coded to allow inclusion in relevant mortality records. Registration of both residency and death is mandated under the Basic Residential Register Law and Family Registry Law, respectively, and the registries are considered complete.

The primary outcomes were all-cause, NCD, cancer, CVD (heart disease, or cerebrovascular disease) mortality. Death from injury, poisoning and certain other consequences of external causes (S00-T98) were excluded from all-cause mortality and censored. Causes of death were confirmed using death certificates and defined based on the International Classification of Disease, 10th revision [23] with the following criteria: cancer (C00–C97), CVD (I00–I99), heart disease (I20–I52), and cerebrovascular disease (I60–I69). NCD mortality further included mortality from diabetes (E10–E14) and respiratory diseases excluding infection (J30–J98), in addition to cancer and CVD mortality.

### Statistical analysis

This population included 17,727 deaths from all causes, 12,860 from NCD, 6921 from cancer (including 852 male and 310 female deaths from esophageal or stomach cancer, accounting for 20% and 12% of total cancer deaths among men and women, respectively), and 4745 deaths from CVD (2438 deaths from coronary artery disease and 1846 from cerebrovascular disease). Participants were followed up until their death, the date they moved residence outside the study area, or the last day of follow-up, whichever occurred first. Participants lost to follow-up or who emigrated during the follow-up period were censored at the last confirmed date of presence in the study area. The mean (SD) years of follow-up for men and women was 18.5 (5.7) and 19.6 (4.8) years, respectively. A total of 1,587,901 person-years were accrued in this analysis. Further, premature NCD mortality was defined as death before age 70 y [9,24]. For analysis of premature NCD death, participants aged ≥70 y at the starting point were excluded and those aged ≥70 y at death were censored [25]. Accordingly, 3555 premature deaths from NCD were identified.

Hazard ratios and 95% CIs were calculated for the categories of energy-adjusted sodium and potassium consumption in quintiles for men and women separately, with the lowest consumption category as reference, using Cox proportional hazards models with adjustment for potential confounding variables according to the SAS PHREG procedure (SAS software, version 9.1; SAS Institute). Energy adjustment of sodium and potassium consumption was done using a residual model for men and women separately [26]. Sodium-to-potassium intake ratio was calculated without energy adjustment [27] in moles per mole.

We conducted the initial analyses by adjusting for age at the starting point (continuous) and public health center area. Linear trends across quintiles of intake were tested using the median consumption for each quintile as an ordinal variable. In the multivariate model, we further adjusted for body mass index in kg/m^2^ (<19, 19–22.9, 23–24.9, 25–26.9, and ≥27); physical activity in metabolic equivalent task-hours per day (<30, 30–34.9, 35–39.9, and ≥40); smoking status (never, past, current <20, and current ≥20 cigarette/d); alcohol consumption (none, occasional, and 1–149, 150–299, 300–449, and ≥450 g of ethanol/wk); marital status (married: yes/no) [28]; living alone (yes/no) [29]; and quintiles of total energy, coffee, green tea, SFA, n–3 PUFA, potassium (for the analysis of sodium) or sodium (for analysis of potassium), screening examination (gastric photofluorography, gastrointestinal endoscopy, fecal occult blood test, barium enema, and colonoscopy), and medication use (hypertension). For missing values of these covariates (smoking status, alcohol drinking, and physical activity), 1782 (4.8%) men and 3621 (7.9%) women were excluded from multivariate analysis; however, it should be noted that analysis with missing values supplemented as dummy variables did not substantially change the results. We conducted further analysis after excluding cases diagnosed within 3 y after the starting point of this study to account for the reversal of cause and effect. To account for the influence of socioeconomic conditions, we performed an additional analysis limited to cohort I, which has information on educational background [30]. Further, with regard to premature NCD mortality, sensitivity analyses were conducted to censor survivors aged ≥70 y during the follow-up period. Moreover, further analyses were conducted by excluding those on hypertensive medications and by mixing men and women. In this article, all *P* values are 2-sided, and statistical significance was determined at the *P* < 0.05 level.

## Results

Sodium intake ranged from a median value of 3101 and 3169 mg (∼8 g salt-equivalent/d) in the lowest quintile for men and women to 6956 and 6752 mg (∼17.5 g) in the highest for men and women, respectively. Potassium intake was higher for women than for men in all categories, and sodium-to-potassium ratio was lower for women than for men in all categories. The difference between the highest compared with lowest quintile groups in median sodium-to-potassium ratio was 2.1-fold, regardless of gender. Participants with higher sodium or potassium consumption were slightly older, in contrast to those with higher sodium-to-potassium ratio. Table 1 show characteristics according to quintile of consumption for men and women, respectively: participants with higher sodium-to-potassium intake ratio were more likely to be obese, current smokers, and heavy drinkers; less likely to consume fruit and vegetables, coffee, or green tea; and less likely to have undergone cancer screening. Higher sodium-to-potassium intake ratio was not associated with physical activity level.

**TABLE 1:** Characteristics of participants by quintiles of sodium and potassium intake and ratio of sodium-to-potassium intake: the JPHC Study, 1995 and 1998 (*N* = 83,048).

	Men (*n* = 37,219)						Women (*n* = 45,829)					
	Sodium		Potassium		Sodium:potassium ratio		Sodium		Potassium		Sodium:potassium ratio	
	Q1	Q5	Q1	Q5	Q1	Q5	Q1	Q5	Q1	Q5	Q1	Q5
No. of participants	7443	7444	7443	7444	7443	7444	9165	9166	9165	9166	9165	9166
Sodium (mg/d), median^1^	3101	6956	3705	5848	3546	6101	3169	6752	3961	5577	3466	6083
Potassium (mg/d), median^1^	2162	3173	1867	3646	3010	2331	2572	3324	2159	3833	3230	2573
Sodium:potassium ratio (mol/mol), median	2.34	3.83	3.46	2.65	2.09	4.32	2.03	3.56	3.20	2.41	1.88	3.92
Age (y), mean ± SD	55.0 ± 7.8	57.4 ± 7.7	54.5 ± 7.4	58.4 ± 7.9	56.9 ± 8.1	55.7 ± 7.4	55.9 ± 8.3	57.5 ± 7.5	55.2 ± 7.9	57.9 ± 7.6	57.0 ± 8.1	56.5 ± 7.6
Physical activity (MET-h/d), mean ± SD)	32.6 ± 6.8	33.0 ± 6.9	33.1 ± 7.0	32.6 ± 6.7	32.2 ± 6.6	33.2 ± 7.0	31.7 ± 5.7	32.2 ± 5.9	31.8 ± 5.9	32.2 ± 5.7	32.0 ± 5.7	32.0 ± 6.0
Obesity: BMI ≥ 27 (%)	11.4	12.4	11.7	11.3	10.7	12.3	11.4	14.1	13.9	12.8	10.9	14.6
Past smoker (%)	17.4	17.0	14.7	18.9	18.6	15.2	1.4	0.9	1.5	0.8	1.0	1.1
Current smoker (%)	48.6	47.8	56.6	37.8	38.5	54.4	7.5	5.6	8.7	4.7	5.6	6.6
Heavy drinker: ≥300 g ethanol/w) (%)	45.0	18.9	57.0	10.8	23.8	38.1	2.8	0.3	3.4	0.2	1.2	1.1
Gastric photofluorography (%)	36.8	37.2	32.4	41.7	42.0	33.1	33.5	35.6	29.3	38.2	36.5	31.1
Gastrointestinal endoscopy (%)	15.7	19.4	13.7	21.1	18.8	16.8	13.6	16.3	11.2	17.3	15.8	13.3
Fecal occult blood test (%)	25.3	26.8	20.9	32.4	31.3	22.7	24.4	27.9	19.3	31.9	28.6	22.0
Barium enema (%)	5.5	8.4	5.2	8.6	7.0	7.0	4.9	7.3	5.4	7.0	5.3	6.4
Colonoscopy (%)	5.8	8.5	5.4	9.1	7.6	6.9	5.0	7.5	4.6	7.2	5.7	6.2
Married: yes (%)	80.6	81.5	75.9	86.2	85.1	77.4	70.9	70.4	67.5	74.0	73.2	67.4
Live alone: yes (%)	6.0	2.7	6.1	2.4	4.5	3.7	9.0	5.0	6.4	7.0	9.3	4.7
Hypertension: medication use (%)	16.6	16.8	15.3	18.6	18.6	15.7	18.4	18.0	15.8	20.1	20.7	16.7
Total energy (kcal/d), median	2054	2035	2077	2053	2032	2075	1754	1759	1744	1767	1783	1747
SFA (g/d), median^1^	14.90	16.92	12.64	18.65	18.18	14.72	18.88	16.18	17.08	17.41	19.08	16.27
n–3 PUFA (g/d), median^1^	1.81	3.05	1.77	3.05	2.26	2.42	2.05	3.00	2.07	2.99	2.36	2.59
Vegetables—excluding pickled vegetable (g/d), median^1^	91	209	76	257	153	122	129	238	107	298	189	155
Fruit (g/d), median^1^	103	175	64	261	192	95	192	238	125	333	279	160
Coffee (g/d), median^1^	129	84	98	94	129	78	103	46	91	52	85	56
Green tea (g/d), median^1^	78	186	50	289	163	88	132	234	54	364	263	91

Abbreviations: MET-h, metabolic equivalent task hours.

1 Dietary intakes other than sodium-to-potassium ratio were adjusted for total energy by residual method.

As shown in Table 2, a statistically significant association between higher sodium intake and increased risk for all-cause mortality was shown in men. In contrast, no association was found for women. Higher sodium intake was significantly associated with cerebrovascular disease–related mortality among women only on exclusion of deaths in the first 3 y (HR: 1.33; 95% CI: 1.00, 1.77 for the highest compared with the lowest; *P*-trend = 0.01; data not shown in the table). NCD death was slightly associated with linear trend only among men. Furthermore, higher sodium intake was associated with premature NCD among men only. These results for outcomes were not substantially changed when deaths in the first 3 y were excluded (data not shown) for both sexes, other than for cerebrovascular disease among women.

**TABLE 2:** Hazard ratios and 95% CIs for all-cause and cause-specific mortality by quintile of sodium consumption, the JPHC Study, 1995 and 1998–2018 (*n* = 37,219 men and 45,829 women).

		Men					*P*-trend^1^	Women					*P*-trend^1^
		Q1	Q2	Q3	Q4	Q5		Q1	Q2	Q3	Q4	Q5	
All-cause death	No. of participants	7443	7444	7444	7444	7444		9165	9166	9166	9166	9166	
	Person, years	133,900	137,554	139,512	139,011	138,138		170,230	177,054	180,899	185,083	186,521	
	Cases	1868	1886	1971	2169	2452		1407	1330	1443	1505	1696	
	ASR	1621	1564	1549	1640	1728		870	816	863	868	912	
	HR1 (95% CI)^2^	1.0 (ref)	0.91 (0.86, 0.98)	0.89 (0.84, 0.95)	0.93 (0.87, 0.99)	0.97 (0.91, 1.03)	*0.95*	1.0 (ref)	0.88 (0.82, 0.95)	0.91 (0.84, 0.98)	0.89 (0.82, 0.96)	0.91 (0.84, 0.98)	0.06
	HR2 (95% CI)^3^	1.0 (ref)	1.01 (0.94, 1.08)	1.02 (0.95, 1.09)	1.09 (1.01, 1.17)	1.11 (1.03, 1.20)	<0.01	1.0 (ref)	0.93 (0.86, 1.01)	0.96 (0.88, 1.04)	0.93 (0.86, 1.02)	0.94 (0.86, 1.03)	0.27
NCDs	Cases	1373	1407	1511	1645	1799		928	925	1035	1072	1165	
	ASR	1184	1154	1179	1228	1264		574	562	615	608	621	
	HR1 (95% CI)^2^	1.0 (ref)	0.92 (0.86, 0.99)	0.92 (0.86, 0.995)	0.95 (0.89, 1.03)	0.96 (0.90, 1.04)	*0.82*	1.0 (ref)	0.92 (0.84, 1.01)	0.97 (0.89, 1.06)	0.94 (0.86, 1.03)	0.93 (0.85, 1.02)	0.24
	HR2 (95% CI)^3^	1.0 (ref)	1.00 (0.93, 1.09)	1.04 (0.95, 1.12)	1.10 (1.01, 1.19)	1.08 (0.99, 1.18)	0.03	1.0 (ref)	0.96 (0.87, 1.06)	1.00 (0.91, 1.11)	0.96 (0.87, 1.07)	0.92 (0.83, 1.02)	0.13
Premature NCDs^4^	No. of participants	6698	7050	7002	6989	6859		8392	8520	8543	8597	8517	
	Person, years	128,055	132,452	133,523	132,960	130,210		158,219	166,581	171,032	175,975	176,153	
	Cases	453	456	445	494	465		253	256	240	233	260	
	ASR	345	338	328	369	357		155	150	138	132	151	
	HR2 (95% CI)^3^	1.0 (ref)	1.13 (0.98, 1.29)	1.13 (0.97, 1.30)	1.31 (1.12, 1.52)	1.25 (1.06, 1.47)	<0.01	1.0 (ref)	1.04 (0.87, 1.26)	0.94 (0.77, 1.14)	0.88 (0.72, 1.08)	0.97 (0.78, 1.21)	0.47
Cancer	Cases	753	820	841	920	971		468	497	545	515	591	
	ASR	639	655	647	677	681		286	296	316	286	315	
	HR1 (95% CI)^2^	1.0 (ref)	0.99 (0.89, 1.09)	0.95 (0.86, 1.05)	0.99 (0.90, 1.10)	0.98 (0.89, 1.08)	*0.85*	1.0 (ref)	0.98 (0.86, 1.11)	1.02 (0.90, 1.15)	0.90 (0.80, 1.03)	0.96 (0.85, 1.09)	0.34
	HR2 (95% CI)^3^	1.0 (ref)	1.08 (0.97, 1.20)	1.08 (0.97, 1.20)	1.15 (1.02, 1.28)	1.12 (0.99, 1.26)	*0.07*	1.0 (ref)	1.02 (0.89, 1.16)	1.02 (0.89, 1.17)	0.89 (0.77, 1.03)	0.92 (0.79, 1.07)	0.09
CVD	Cases	493	455	522	556	621		384	357	402	476	479	
	ASR	433	383	411	420	436		240	222	245	274	257	
	HR1 (95% CI)^2^	1.0 (ref)	0.83 (0.73, 0.94)	0.88 (0.78, 0.99)	0.88 (0.78, 0.995)	0.90 (0.80, 1.01)	*0.40*	1.0 (ref)	0.86 (0.75, 0.996)	0.91 (0.79, 1.05)	1.01 (0.88, 1.15)	0.91 (0.79, 1.04)	0.70
	HR2 (95% CI)^3^	1.0 (ref)	0.91 (0.79, 1.05)	1.00 (0.87, 1.15)	1.05 (0.91, 1.21)	1.03 (0.88, 1.19)	*0.31*	1.0 (ref)	0.90 (0.77, 1.05)	0.96 (0.82, 1.12)	1.08 (0.93, 1.27)	0.91 (0.77, 1.08)	0.75
Heart disease	Cases	257	248	270	283	322		212	181	200	249	216	
	ASR	230	211	212	213	226		133	114	123	146	116	
	HR1 (95% CI)^2^	1.0 (ref)	0.87 (0.73, 1.04)	0.88 (0.74, 1.05)	0.87 (0.74, 1.04)	0.91 (0.77, 1.08)	*0.46*	1.0 (ref)	0.81 (0.66, 0.99)	0.85 (0.70, 1.03)	1.00 (0.83, 1.21)	0.78 (0.64, 0.95)	0.13
	HR2 (95% CI)^3^	1.0 (ref)	1.00 (0.83, 1.20)	1.04 (0.86, 1.27)	1.07 (0.87, 1.30)	1.08 (0.87, 1.33)	*0.40*	1.0 (ref)	0.80 (0.65, 0.997)	0.85 (0.68, 1.05)	1.03 (0.83, 1.27)	0.73 (0.57, 0.92)	0.09
Cerebrovascular disease	Cases	182	168	206	212	241		132	136	152	198	219	
	ASR	156	138	162	160	169		82	83	91	111	116	
	HR1 (95% CI)^2^	1.0 (ref)	0.81 (0.66, 1.00)	0.92 (0.75, 1.13)	0.89 (0.73, 1.09)	0.92 (0.76, 1.12)	*0.82*	1.0 (ref)	0.93 (0.73, 1.18)	0.96 (0.76, 1.21)	1.14 (0.91, 1.43)	1.13 (0.90, 1.41)	0.06
	HR2 (95% CI)^3^	1.0 (ref)	0.92 (0.73, 1.15)	1.08 (0.86, 1.35)	1.12 (0.89, 1.41)	1.09 (0.86, 1.40)	*0.24*	1.0 (ref)	1.05 (0.81, 1.37)	1.11 (0.85, 1.45)	1.35 (1.04, 1.75)	1.28 (0.97, 1.69)	0.03

Abbreviations: ASR, age standardized mortality rate (per 100,000 person-years); HR, hazard ratio.

1 Linear trends across quintiles of sodium intake were tested using the median consumption for each quintile as an ordinal variable.

2 HR1: adjusted for age (continuous) and public health center area.

3 HR2: further adjusted for BMI in kg/m^2^ (<19, 19–22.9, 23–24.9, 25–26.9, and ≥27), physical activity in metabolic equivalent task-hours per day (<30, 30–34.9, 35–39.9, and ≥40), smoking status (never, past, current <20, and current ≥20 cigarette/d), alcohol consumption (none, occasional, and 1–149, 150–299, 300–449, and ≥450 g ethanol/wk), marital status (married: yes/no), live alone (yes/no), quintile of energy intake, coffee, green tea, SFA, n–3 PUFA, and potassium, screening examination (gastric photofluorography, gastrointestinal endoscopy, fecal occult blood test, barium enema, and colonoscopy), and medication use (hypertension).

4 For analysis of premature NCD death, participants aged ≥70 y at baseline were excluded, and data for those aged ≥70 y at death were treated as censored observations.

On the contrary, as shown in Table 3, an inverse association was found between potassium intake and all-cause death and NCD risk. Further, a significant inverse association was found for CVD death. In contrast, no statistically significant inverse association was found for premature NCD death. Potassium was not significantly associated with cancer mortality in either sex. With regard to women, no association was found for any outcome on multivariate analysis. Additionally, on analysis for CVD death according to deciles, the inverse association was not changed among men (HR: 0.78; 95% CI: 0.63, 0.97 for the highest decile compared with the lowest; *P*-trend < 0.01; data not shown in the table), whereas the magnitude of relative risk negatively increased for women, albeit without statistical significance (HR: 0.87; 95% CI: 0.69, 1.09; *P*-trend = 0.59; data not shown in the table). Analysis that excluded deaths in the first 3 y did not substantially change the results for any outcome (data not shown) for both sexes.

**TABLE 3:** Hazard ratios and 95% CIs for all-cause and cause-specific mortality according to quintile of potassium consumption, the JPHC Study, 1995 and 1998–2018 (*n* = 37,219 men and 45,829 women).

		Men					*P*-trend^1^	Women					*P*-trend^1^
		Q1	Q2	Q3	Q4	Q5		Q1	Q2	Q3	Q4	Q5	
All-cause death	No. of participants	7443	7444	7444	7444	7444		9165	9166	9166	9166	9166	
	Person-years	136,467	138,253	138,259	138,910	136,225		178,940	179,740	180,470	179,822	180,814	
	Cases	2097	1908	1958	2051	2332		1449	1384	1453	1469	1626	
	ASR	1892	1650	1597	1522	1503		937	883	864	819	844	
	HR1 (95% CI)^2^	1.0 (ref)	0.85 (0.80, 0.90)	0.80 (0.75, 0.85)	0.74 (0.70, 0.79)	0.73 (0.69, 0.77)	<0.01	1.0 (ref)	0.92 (0.85, 0.99)	0.89 (0.82, 0.95)	0.84 (0.78, 0.9)	0.85 (0.79, 0.92)	<0.01
	HR2 (95% CI)^3^	1.0 (ref)	0.94 (0.88, 1.01)	0.91 (0.85, 0.98)	0.89 (0.82, 0.96)	0.89 (0.81, 0.96)	<0.01	1.0 (ref)	1.00 (0.92, 1.09)	1.01 (0.92, 1.09)	0.99 (0.91, 1.08)	1.01 (0.92, 1.10)	0.90
NCDs	Cases	1541	1478	1457	1562	1697		985	959	1002	1032	1147	
	ASR	1370	1255	1177	1154	1094		628	604	587	573	597	
	HR1 (95% CI)^2^	1.0 (ref)	0.89 (0.83, 0.96)	0.81 (0.75, 0.87)	0.78 (0.73, 0.84)	0.73 (0.68, 0.79)	<0.01	1.0 (ref)	0.93 (0.85, 1.02)	0.90 (0.82, 0.98)	0.87 (0.80, 0.96)	0.90 (0.82, 0.98)	0.01
	HR2 (95% CI)^3^	1.0 (ref)	0.98 (0.90, 1.06)	0.92 (0.84, 0.998)	0.92 (0.84, 1.01)	0.89 (0.81, 0.98)	0.02	1.0 (ref)	1.00 (0.90, 1.10)	1.00 (0.90, 1.11)	0.99 (0.90, 1.10)	1.03 (0.93, 1.15)	0.56
Premature NCDs^4^	No. of participants	7130	7130	7032	6932	6674		8614	8584	8532	8458	8381	
	Person-years	132,389	134,098	132,848	131,981	125,885		170,251	170,533	170,444	168,539	168,193	
	Cases	559	503	455	420	376		297	249	253	214	229	
	ASR	651	616	594	576	551		267	280	265	250	275	
	HR2 (95% CI)^3^	1.0 (ref)	1.02 (0.89, 1.16)	0.97 (0.83, 1.12)	0.94 (0.80, 1.10)	0.94 (0.79, 1.12)	0.36	1.0 (ref)	0.98 (0.81, 1.17)	1.02 (0.84, 1.23)	0.95 (0.78, 1.16)	1.00 (0.81, 1.24)	0.96
Cancer	Cases	836	832	821	880	936		478	508	517	521	592	
	ASR	723	690	639	645	612		290	307	296	288	312	
	HR1 (95% CI)^2^	1.0 (ref)	0.93 (0.85, 1.02)	0.86 (0.78, 0.94)	0.84 (0.76, 0.92)	0.79 (0.72, 0.86)	<0.01	1.0 (ref)	1.02 (0.90, 1.16)	0.98 (0.86, 1.11)	0.94 (0.83, 1.06)	1.00 (0.88, 1.13)	0.69
	HR2 (95% CI)^3^	1.0 (ref)	1.04 (0.94, 1.16)	1.00 (0.89, 1.12)	1.03 (0.91, 1.16)	1.01 (0.89, 1.15)	0.97	1.0 (ref)	1.06 (0.93, 1.22)	1.05 (0.91, 1.21)	1.05 (0.91, 1.21)	1.14 (0.98, 1.32)	0.13
CVD	Cases	576	510	472	520	569		414	390	406	429	459	
	ASR	529	441	390	387	363		276	256	244	239	236	
	HR1 (95% CI)^2^	1.0 (ref)	0.82 (0.73, 0.93)	0.69 (0.62, 0.79)	0.68 (0.60, 0.76)	0.63 (0.56, 0.71)	<0.01	1.0 (ref)	0.90 (0.78, 1.03)	0.85 (0.74, 0.97)	0.84 (0.73, 0.96)	0.81 (0.71, 0.93)	<0.01
	HR2 (95% CI)^3^	1.0 (ref)	0.89 (0.78, 1.02)	0.78 (0.67, 0.90)	0.80 (0.69, 0.94)	0.78 (0.66, 0.92)	0.01	1.0 (ref)	0.98 (0.84, 1.14)	0.98 (0.84, 1.15)	0.97 (0.83, 1.14)	0.97 (0.82, 1.15)	0.76
Heart disease	Cases	303	271	251	261	294		206	212	197	222	221	
	ASR	286	236	210	194	189		138	141	120	124	113	
	HR1 (95% CI)^2^	1.0 (ref)	0.84 (0.71, 0.98)	0.71 (0.60, 0.83)	0.65 (0.55, 0.77)	0.63 (0.53, 0.74)	<0.01	1.0 (ref)	0.99 (0.82, 1.20)	0.84 (0.69, 1.02)	0.89 (0.73, 1.07)	0.80 (0.66, 0.97)	0.01
	HR2 (95% CI)^3^	1.0 (ref)	0.89 (0.74, 1.07)	0.75 (0.61, 0.92)	0.75 (0.61, 0.93)	0.74 (0.59, 0.93)	0.01	1.0 (ref)	1.08 (0.88, 1.34)	1.00 (0.80, 1.25)	1.08 (0.86, 1.36)	1.07 (0.85, 1.36)	0.60
Cerebrovascular disease	Cases	232	186	173	199	219		160	142	176	164	195	
	ASR	205	158	142	150	139		105	93	104	91	102	
	HR1 (95% CI)^2^	1.0 (ref)	0.74 (0.61, 0.90)	0.63 (0.52, 0.77)	0.64 (0.53, 0.78)	0.61 (0.50, 0.73)	<0.01	1.0 (ref)	0.83 (0.66, 1.04)	0.93 (0.75, 1.15)	0.81 (0.65, 1.01)	0.87 (0.70, 1.08)	0.27
	HR2 (95% CI)^3^	1.0 (ref)	0.82 (0.66, 1.02)	0.76 (0.6, 0.96)	0.79 (0.62, 1.01)	0.79 (0.61, 1.03)	0.17	1.0 (ref)	0.90 (0.70, 1.15)	1.00 (0.78, 1.28)	0.85 (0.65, 1.10)	0.87 (0.66, 1.13)	0.27

Abbreviations: ASR, age standardized mortality rate (per 100,000 person-years); HR, hazard ratio.

1 Linear trends across quintiles of potassium intake were tested using the median consumption for each quintile as an ordinal variable.

2 HR1: adjusted for age (continuous) and public health center area.

3 HR2: further adjusted for body mass index in kg/m^2^ (<19, 19–22.9, 23–24.9, 25–26.9, and ≥27), physical activity in metabolic equivalent task-hours/d (<30, 30–34.9, 35–39.9, and ≥40), smoking status (never, past, current <20, and current ≥20 cigarette/d), alcohol consumption (none, occasional, and 1–149, 150–299, 300–449, and ≥450 g ethanol/wk), marital status (married: yes/no), living alone (yes/no), quintile of energy intake, coffee, green tea, SFA, n–3 PUFA, and sodium, screening examination (gastric photofluorography, gastrointestinal endoscopy, fecal occult blood test, barium enema, and colonoscopy), and medication use (hypertension).

4 For analysis of premature NCD death, participants aged ≥70 y at baseline were excluded, and data for those aged ≥70 y at death were treated as censored observations.

With regard to sodium-to-potassium ratio, as shown in Table 4, significant associations were found with all outcomes among men. HRs were stronger than for sodium with adjustment for potassium in the multivariate models. In women, the association between sodium-to-potassium intake ratio and cerebrovascular disease did not strengthen above those of sodium intake on exclusion of deaths in the first 3 y (HR: 1.21; 95% CI: 0.94, 1.55 for the highest compared with the lowest; *P*-trend = 0.04; data not shown in the table). Analysis excluding deaths in the first 3 y did not substantially change the results for any outcome (data not shown) for both sexes, including cerebrovascular disease, as noted earlier.

**TABLE 4:** Hazard ratios and 95% CIs for all-cause and cause-specific mortality by quintile of sodium-to-potassium consumption ratio, the JPHC Study, 1995 and 1998–2018 (*n* = 37,219 men and 45,829 women).

		Men					*P*-trend^1^	Women					*P*-trend^1^
		Q1	Q2	Q3	Q4	Q5		Q1	Q2	Q3	Q4	Q5	
All-cause death	No. of participants	7443	7444	7444	7444	7444		9165	9166	9166	9166	9166	
	Person-years	134,300	136,840	138,641	139,988	138,346		171,125	177,207	180,897	184,662	185,895	
	Cases	1905	1927	2016	2103	2395		1461	1360	1478	1410	1672	
	ASR	1391	1509	1594	1679	1952		823	802	885	851	978	
	HR1 (95% CI)^2^	1.0 (ref)	1.08 (1.01, 1.15)	1.11 (1.04, 1.18)	1.17 (1.1, 1.25)	1.39 (1.31, 1.48)	<0.01	1.0 (ref)	0.94 (0.87, 1.01)	1.03 (0.95, 1.10)	0.96 (0.89, 1.03)	1.09 (1.01, 1.17)	<0.01
	HR2 (95% CI)^3^	1.0 (ref)	1.05 (0.98, 1.12)	1.06 (0.99, 1.13)	1.06 (0.996, 1.14)	1.19 (1.11, 1.27)	<0.01	1.0 (ref)	0.96 (0.88, 1.04)	1.00 (0.92, 1.08)	0.93 (0.86, 1.01)	0.99 (0.92, 1.08)	0.76
NCDs	Cases	1373	1476	1512	1571	1803		990	939	1042	1006	1148	
	ASR	1004	1147	1187	1241	1450		560	552	618	596	659	
	HR1 (95% CI)^2^	1.0 (ref)	1.13 (1.05, 1.22)	1.14 (1.06, 1.23)	1.20 (1.11, 1.29)	1.42 (1.32, 1.53)	<0.01	1.0 (ref)	0.95 (0.87, 1.04)	1.04 (0.96, 1.14)	0.98 (0.90, 1.07)	1.07 (0.98, 1.17)	0.07
	HR2 (95% CI)^3^	1.0 (ref)	1.11 (1.02, 1.19)	1.08 (1.00, 1.17)	1.08 (1.00, 1.17)	1.22 (1.13, 1.32)	<0.01	1.0 (ref)	0.96 (0.87, 1.05)	0.99 (0.90, 1.09)	0.94 (0.85, 1.03)	0.97 (0.88, 1.07)	0.52
Premature NCDs^4^	No. of participants	6791	6960	7005	7041	7101		8317	8453	8530	8662	8607	
	Person-years	125,646	130,498	132,598	134,478	133,980		157,729	165,752	170,756	176,603	177,120	
	Cases	369	433	408	509	594		220	237	250	269	266	
	ASR	296	329	303	371	436		140	141	144	151	150	
	HR2 (95% CI)^3^	1.0 (ref)	1.11 (0.96, 1.28)	1.00 (0.87, 1.16)	1.16 (1.01, 1.34)	1.27 (1.10, 1.46)	<0.01	1.0 (ref)	0.99 (0.82, 1.19)	0.96 (0.79, 1.16)	0.99 (0.82, 1.20)	0.90 (0.74, 1.10)	0.32
Cancer	Cases	776	870	815	868	976		490	520	554	511	541	
	ASR	568	670	629	662	768		280	302	320	295	302	
	HR1 (95% CI)^2^	1.0 (ref)	1.16 (1.06, 1.28)	1.07 (0.97, 1.18)	1.15 (1.04, 1.27)	1.33 (1.21, 1.47)	<0.01	1.0 (ref)	1.04 (0.92, 1.18)	1.09 (0.96, 1.23)	0.98 (0.86, 1.11)	1.01 (0.89, 1.14)	0.65
	HR2 (95% CI)^3^	1.0 (ref)	1.14 (1.03, 1.26)	1.03 (0.93, 1.15)	1.05 (0.95, 1.16)	1.18 (1.07, 1.31)	0.02	1.0 (ref)	1.04 (0.91, 1.18)	1.02 (0.89, 1.16)	0.91 (0.80, 1.05)	0.91 (0.79, 1.04)	0.04
CVD	Cases	465	458	533	541	650		417	347	403	420	511	
	ASR	340	359	423	441	529		234	207	245	255	301	
	HR1 (95% CI)^2^	1.0 (ref)	1.05 (0.92, 1.20)	1.20 (1.06, 1.36)	1.23 (1.08, 1.39)	1.53 (1.36, 1.73)	<0.01	1.0 (ref)	0.84 (0.73, 0.97)	0.98 (0.86, 1.13)	1.00 (0.87, 1.15)	1.16 (1.01, 1.32)	<0.01
	HR2 (95% CI)^3^	1.0 (ref)	1.00 (0.88, 1.15)	1.10 (0.97, 1.26)	1.08 (0.95, 1.24)	1.22 (1.07, 1.39)	<0.01	1.0 (ref)	0.87 (0.75, 1.02)	0.96 (0.83, 1.12)	0.97 (0.84, 1.13)	1.04 (0.90, 1.21)	0.25
Heart disease	Cases	249	243	273	281	334		220	176	204	202	256	
	ASR	182	189	220	225	277		123	106	126	123	153	
	HR1 (95% CI)^2^	1.0 (ref)	1.05 (0.88, 1.26)	1.16 (0.98, 1.39)	1.21 (1.02, 1.44)	1.49 (1.26, 1.76)	<0.01	1.0 (ref)	0.83 (0.68, 1.01)	0.98 (0.81, 1.19)	0.96 (0.79, 1.17)	1.16 (0.96, 1.39)	0.03
	HR2 (95% CI)^3^	1.0 (ref)	1.03 (0.85, 1.23)	1.09 (0.91, 1.30)	1.10 (0.92, 1.32)	1.24 (1.03, 1.49)	0.02	1.0 (ref)	0.84 (0.68, 1.04)	0.92 (0.74, 1.13)	0.90 (0.73, 1.11)	0.99 (0.81, 1.22)	0.77
Cerebrovascular disease	Cases	161	170	198	213	267		153	134	162	178	210	
	ASR	118	133	155	178	213		86	79	97	109	120	
	HR1 (95% CI)^2^	1.0 (ref)	1.11 (0.89, 1.38)	1.26 (1.02, 1.55)	1.36 (1.10, 1.67)	1.76 (1.44, 2.15)	<0.01	1.0 (ref)	0.86 (0.68, 1.09)	1.03 (0.83, 1.29)	1.09 (0.88, 1.36)	1.22 (0.99, 1.52)	<0.01
	HR2 (95% CI)^3^	1.0 (ref)	1.04 (0.83, 1.30)	1.15 (0.92, 1.43)	1.16 (0.93, 1.44)	1.32 (1.06, 1.64)	<0.01	1.0 (ref)	0.94 (0.73, 1.21)	1.11 (0.87, 1.41)	1.14 (0.89, 1.46)	1.21 (0.95, 1.55)	0.04

Abbreviations: ASR, age standardized mortality rate (per 100,000 person-years); HR, hazard ratio.

1 Linear trends across quintiles of sodium-to-potassium ratio were tested by using the median consumption for each quintile as an ordinal variable.

2 HR1: adjusted for age (continuous), and public health center area.

3 HR2: further adjusted for body mass index in kg/m^2^ (<19, 19–22.9, 23–24.9, 25–26.9, and ≥27), physical activity in metabolic equivalent task-hours/d (<30, 30–34.9, 35–39.9, and ≥40), smoking status (never, past, current <20, and current ≥20 cigarette/d), alcohol consumption (none, occasional, and 1–149, 150–299, 300–449, and ≥450 g ethanol/wk), marital status (married: yes/no), living alone (yes/no), quintile of energy intake, coffee, green tea, SFA, and n–3 PUFA, screening examination (gastric photofluorography, gastrointestinal endoscopy, fecal occult blood test, barium enema, and colonoscopy), and medication use (hypertension).

4 For analysis of premature NCD death, participants aged ≥70 y at baseline were excluded, and data for those aged ≥70 y at death were treated as censored observations.

In an additional analysis considering socioeconomic conditions limited to cohort I, either results of death from all-cause or NCD were not substantially changed for both sexes, with corresponding HRs among men of 1.19 (95% CI: 1.04, 1.37; *P*-trend < 0.01) and 1.16 (95% CI: 1.00, 1.36; *P*-trend = 0.05) for sodium intake and 1.23 (95% CI: 1.08, 1.39; *P*-trend < 0.01) and 1.26 (95% CI: 1.09, 1.45; *P*-trend < 0.01) for sodium-to-potassium ratio, respectively. Moreover, with regard to premature NCD mortality, sensitivity analyses were conducted to censor survivors aged 70 y or older during the follow-up period. The results were not substantially changed, as follows: HRs for the highest compared with the lowest values for men and women were 1.25 (95% CI: 1.06, 1.47; *P*-trend < 0.01) and 0.98 (95% CI: 0.79, 1.22; *P*-trend = 0.54), respectively, for sodium; 0.95 (95% CI: 0.80, 1.14; *P*-trend = 0.44) and 0.98 (95% CI: 0.79, 1.22; *P*-trend = 0.80), respectively, for potassium; and 1.26 (95% CI: 1.09, 1.45; *P*-trend < 0.001) and 0.92 (95% CI: 0.75, 1.11; *P*-trend = 0.41), respectively, for sodium-to-potassium ratio. We conducted further analyses by excluding hypertensive participants (Supplemental Table 1). The results were not substantially changed, although the associations between intake of sodium or sodium-to-potassium ratio and cerebrovascular disease were slightly strengthened: corresponding HRs for the highest intake compared with the lowest (95% CIs) were 1.17 (95% CI: 0.88, 1.56; *P*-trend = 0.11) and 1.58 (95% CI: 1.11, 2.25; *P*-trend = 0.01) for men and women, respectively, for sodium, and 1.42 (95% CI: 1.10, 1.83; *P*-trend = 0.01) and 1.39 (95% CI: 1.03, 1.89; *P*-trend < 0.01) for men and women, respectively, for sodium-to-potassium ratio. Furthermore, in analyses in men and women combined and adjusted for sex, all results for all-cause, NCD, CVD, and cerebrovascular disease–related mortality remained statistically significant (Supplemental Table 2), similar to the results shown in men, with corresponding HRs for sodium-to-potassium ratio of 1.09 (95% CI: 1.03, 1.14; *P*-trend < 0.001), 1.09 (95% CI: 1.03, 1.16; *P*-trend = 0.01), 1.11 (95% CI: 1.01, 1.23; *P*-trend = 0.01), and 1.27 (95% CI: 1.08, 1.50; *P*-trend < 0.01), respectively. Furthermore, additional adjustment for sugary drinks [31] made little difference to the results (data not shown). Sensitivity analysis for sodium consumption, which included vegetables and fruit instead of potassium consumption as adjustment factors showed slightly de-attenuated HRs in male cancer–related mortality only (HR for the highest compared with the lowest: 1.13; 95% CI: 1.01, 1.28; *P*-trend = 0.04), and not in female cancer–related mortality (HR: 0.94; 95% CI: 0.81, 1.09; *P*-trend = 0.15). The results for mortality from all cause and NCD did not change (data not shown). A nonlinear association was also examined. A significant association was found only between male potassium intake and mortality from CVD (*P* = 0.04). We also carried out logistic regression analysis because of the possibility that the proportional hazard assumption would not be supported. The results were not substantially changed with regard to either exposure or outcome for both sexes: odd ratios of death from all causes for the highest compared with lowest (95% CIs) intake for men and women were 1.17 (95% CI: 1.06, 1.30; *P*-trend < 0.01) and 0.92 (95% CI: 0.83, 1.03; *P*-trend = 0.24), respectively, for sodium; 0.85 (95% CI: 0.76, 0.95; *P*-trend = 0.01) and 1.01 (95% CI: 0.91, 1.12; *P*-trend = 0.99), respectively, for potassium; and 1.27 (95% CI: 1.16, 1.38; *P*-trend < 0.001) and 0.99 (95% CI: 0.90, 1.09; *P*-trend = 0.81), respectively, for sodium-to-potassium ratio.

## Discussion

We found that sodium intake was significantly associated with increased risk of all-cause mortality and premature mortality from NCD in men and with cerebrovascular disease–related mortality in women. Potassium intake was significantly associated with decreased risk of all-cause, NCD, and CVD mortalities, but not with mortality from cancer, in men. In contrast, potassium intake was not associated with any outcome in women. Increased sodium-to-potassium intake ratio was associated with significantly increased risk for all outcomes in men, including cancer. In contrast, sodium-to-potassium ratio in women showed a significant linear trend for cerebrovascular disease–related mortality only. To our knowledge, this is the first study to examine the association of intake of sodium and sodium-to-potassium ratio with premature NCD deaths.

A previous report from this JPHC study on the association between sodium consumption and CVD and stroke incidence using the same FFQ [18] as this study found a significant positive association for CVD and stroke, reflecting our results for mortality from CVD and cerebrovascular disease among men and women combined. Our results are consistent with that previous report, which used incidence as the outcome. Possible mechanisms of CVD via hypertension due to excessive sodium intake may include homeostatic mechanisms for body fluids, persistent inflammatory conditions, and intestinal flora [32]. Inconsistent with our results, a previous study based on very short-term intervention studies suggested that compensatory mechanisms following acute and significant reductions in salt intake had harmful effects, such as increases in plasma renin activity [6]. The difference in results is probably due to the fact that this study was a long-term observation of a population with a high salt intake. Further, our present use of sodium-to-potassium ratio of intake as exposure and the addition of NCD mortality, particularly premature NCD mortality, as outcome provide additional information not available in the previous study.

Our results for men with very high sodium intake [6,7] are consistent with a meta-analysis that reported a higher hazard of all-cause mortality with high compared with low sodium intake [33]. Our finding of no significant increase in risk for either CVD or cerebrovascular disease–related mortality with sodium but a significant increase with ratio to potassium (and also an association of higher potassium with reduced CVD mortality, including cerebrovascular disease) among men is consistent with previous studies, suggesting an additive role of increased sodium and decreased potassium intakes in increasing CVD risk [13]. In fact, a meta-analysis of prospective studies examining the association between both sodium intake and sodium-to-potassium ratio and risk of cerebrovascular disease reported greater relative risk for sodium-to-potassium ratio than that for sodium alone [12]. These trends remained when exposure was assessed using 24-h urinary excretion and outcome was CVD [13]. The observed stronger associations between sodium (including ratio to potassium intake) and CVD (among men) and cerebrovascular disease (among women), respectively, in analyses which excluded those receiving hypertension treatment, may indicate that the causal inversion (owing to hypertensives’ commitment to salt reduction) has been eliminated. Contradictory to our results, a recent cohort study based on the NHANES in the United States reported that intakes of both sodium and potassium were inversely associated with all-cause mortality [34]. The major reason for the difference in results may be, first, the study used an exposure assessment based on a 24-h recall method over 1 or 2 days, which does not account for intraindividual variability; and second, differences in intake levels in the population (when compared by 24-h urinary sodium excretion) [7].

Contrary to our results, a stratified analysis by gender in a meta-analysis of 5 cohort studies (enrolling 57,537 men and 74,018 women; 2 Japanese cohorts accounted for ≥85% of the total), which examined the association between sodium and CVD mortality showed a slightly stronger hazard for women than that for men [13,35]. Despite the significant association between sodium and cerebrovascular disease–related mortality, this study saw no substantial association between sodium-to-potassium ratio and cerebrovascular disease–related mortality among women, in contrast to men. This attenuated association with sodium-to-potassium ratio over sodium among women may be because the FFQ had lower validity for both sodium and potassium intakes than multiple 24-h urinary excretion in women than men, and in particular, a lower validity for potassium than sodium in women [20]. Further, the beneficial effect of potassium on CVD may have been missed because the intake of potassium in women was sufficient. This is because women had higher intake than men, and the magnitude of the association in men did not increase with increasing intake above the third quintile, whereas HRs for women by decile were somewhat greater than those by quintile.

We observed an association between higher sodium-to-potassium ratio and increased cancer mortality among men only. A joint report from the Continuous Update project by the World Cancer Research Fund/American Institute for Cancer Research 2018 revision noted that salted food is probably a risk-increasing factor for gastric cancer, whereas nonstarchy vegetables or fruit are probably a risk-decreasing factor for aggregated aerodigestive cancers [36]. Sodium may be a proxy for salt intake from these salted foods, although potassium may be a proxy for intake of fruit and vegetables. It is therefore reasonable to consider sodium-to-potassium ratio as an overall indicator of the joint effect of high-salted food and low-fruit and vegetable intake, rather than as a mechanism by which sodium and/or potassium directly affect cancers. These effects may be related to gastrointestinal cancers, particularly esophageal and stomach cancers, for which we previously reported a negative association with vegetables and fruits [37,38] and positive association with salted foods [18]. Moreover, the difference in results between men and women for cancer was likely attributable to the differing percentages of gastrointestinal cancers, with esophageal and stomach cancer–related deaths in this analysis accounting for 20% and 12% of total cancer deaths for men and women, respectively.

Although several prospective studies have examined risk of premature death, only 3 of these examined the association of dietary factors with premature death [[39], [40], [41]]. The Mediterranean diet score showed no significant association with risk of premature all-cause mortality among 17,747 Spaniards [39]. A healthy diet score based on the WCRF/AICR was associated with lower risk of premature all-cause mortality among 264,906 European adults in the European Prospective Investigation into Cancer and Nutrition (EPIC) cohort study [40]. Combined fruit and vegetable consumption was associated with lower risk of premature all-cause mortality in 50,045 Iranians [41]. However, to our knowledge, no prospective study has addressed the association between diet, including sodium intake or sodium-to-potassium ratio, and premature NCD mortality itself as outcomes.

The major strength of this study is its prospective design, which avoided exposure recall bias. Other strengths include definition of the general population as study participants; acceptable response rate to the questionnaire (76.3%) for studies such as this; and negligible proportion of losses to follow-up (*n* = 5202; 6.2%). Our study also has several possible limitations. First, the validity of the FFQ for sodium and potassium was moderate at best (*r* = 0.42, and 0.40, respectively, for men; *r* = 0.30 and 0.24, respectively, for women) compared with multiple 24-h urinary excretion as reference, as described earlier with regard to difference by gender. Some misclassification in the FFQ was unavoidable. Observed associations would have underestimated the true magnitude of the associations, particularly for women. The possibility of misclassification might have been increased by the inability to capture changes over time using a single time point FFQ at baseline. Moreover, although cause of death coding might not be completely accurate, it occurred independently of the exposure information, and might accordingly have biased the hazard ratio toward null [42]. Furthermore, the participants excluded from the analysis were older than those included in the analysis, and accordingly were at higher risk of death and were more likely to have generally eaten a diet with a high salt intake [43]. The exclusion of these participants might therefore have resulted in the underestimation of the association. Second, the variation in sodium-to-potassium ratio was also moderate, with median values in the highest compared with lowest quintile group differing by only 2.1-fold regardless of gender. However, this range is similar to the 2.2-fold difference in a study based on 24-h urinary excretion, which identified positive associations between sodium-to-potassium ratio and CVD [13]. Accordingly, our variation for women was sufficient to detect an association between sodium-to-potassium ratio and CVD risk. Third, we could not consider changes in diet after diagnosis. Although dietary changes in survivors after any cancer diagnosis may not substantially differ from those in people without a cancer diagnosis [44], we cannot guarantee that these results would also be seen in survivors of cardiovascular disease or that there are no differences between men and women.

In conclusion, we found that sodium intake was significantly associated with increased risk of all-cause mortality and premature mortality from NCD in men and with increased risk of cerebrovascular disease–related mortality in women. When intakes are expressed in terms of ratio to potassium intake, these associations, including those for cancers, were strengthened in men, suggesting an additive role of increased sodium intake and decreased potassium intake in increasing all-cause and premature NCD mortality.

## Author contributions

The authors’ responsibilities were as follows — NS: was involved in the design of the study as the principal investigator; ST, NS, AG, TY, KY, HI, M. Iwasaki, and M. Inoue: conducted the survey; RT, MY: conducted the data analysis; RT: drafted the plans for data analyses, drafted the original manuscript, and had primary responsibility for final content; NS, KY: supervised the analysis and preparation of the manuscript; and all authors: were involved in the interpretation of the results and revision of the manuscript and read and approved the final manuscript.

## Data availability

Access to Japan Public Health Centre-based Prospective Study data will be made available upon reasonable request. Follow the instructions at https://epi.ncc.go.jp/en/jphc/805/8155.html.

## Funding

This work was funded by the National Cancer Centre Research and Development Fund (2011–).

## Conflict of interest

The authors report no conflicts of interest.
