# Supplementary material for: Consumption of Sodium and Its Ratio to Potassium in Relation to All-Cause, Cause-Specific, and Premature Noncommunicable Disease Mortality in Middle-Aged Japanese Adults: A Prospective Cohort Study
Source: J Nutr. 2024 Dec 27;155(3):945–56. doi: 10.1016/j.tjnut.2024.12.020 (PMC11934238; doi:10.1016/j.tjnut.2024.12.020)
Supplement: Multimedia component 1 [file mmc1.docx]

Supplemental TABLE 1. Hazard ratios and 95% confidence intervals for all-cause and NCD and CVD mortality by quintile of sodium consumption and sodium-to-potassium ratio among subjects excluding hypertension medication users: the JPHC Study, 1995 and 1998–2018

|  |  | **Men** (n=31,048) | | | | | *trend p*^4)^ |  | **Women** (n= 37,612) | | | | | *trend p*^4)^ |
| --- | --- | --- | --- | --- | --- | --- | --- | --- | --- | --- | --- | --- | --- | --- |
|  |  | **Q1** | **Q2** | **Q3** | **Q4** | **Q5** |  |  | **Q1** | **Q2** | **Q3** | **Q4** | **Q5** |  |
| **Sodium** |  |  |  |  |  |  |  |  |  |  |  |  |  |  |
|  | No. of subjects | 6,206 | 6,216 | 6,211 | 6,219 | 6,196 |  |  | 7,477 | 7,545 | 7,561 | 7,513 | 7,516 |  |
|  | Person-years | 112,705 | 116,135 | 117,837 | 117,453 | 116,528 |  |  | 139,296 | 146,420 | 150,209 | 152,581 | 154,372 |  |
| All-cause death | Cases | 1,427 | 1,434 | 1,488 | 1,652 | 1,882 |  |  | 920 | 934 | 1,014 | 1,059 | 1,227 |  |
|  | ASR^1)^ | 1,456 | 1,391 | 1,373 | 1,473 | 1,564 |  |  | 705 | 690 | 725 | 732 | 780 |  |
|  | HR2 (95% CI)^2,3)^ | 1.0 (ref) | 1.00 (0.93-1.09) | 1.02 (0.94-1.11) | **1.11 (1.03-1.21)** | **1.14 (1.05-1.25)** | ***<.001*** |  | 1.0 (ref) | 0.98 (0.89-1.08) | 1.00 (0.90-1.10) | 0.97 (0.87-1.07) | 0.99 (0.89-1.10) | *0.81* |
| NCD | Cases | 1,044 | 1,089 | 1,155 | 1,250 | 1,382 |  |  | 614 | 672 | 739 | 755 | 838 |  |
|  | ASR^1)^ | 1,058 | 1,047 | 1,059 | 1,101 | 1,144 |  |  | 469 | 492 | 524 | 514 | 530 |  |
|  | HR2 (95% CI)^2,3)^ | 1.0 (ref) | 1.02 (0.93-1.11) | 1.05 (0.96-1.15) | **1.12 (1.01-1.23)** | 1.10 (.997-1.22) | ***0.02*** |  | 1.0 (ref) | 1.04 (0.92-1.17) | 1.05 (0.93-1.18) | 1.00 (0.88-1.13) | 0.95 (0.84-1.09) | *0.24* |
| Premature NCD^5)^ | No. of subjects | 5,905 | 5,966 | 5,916 | 5,927 | 5,805 |  |  | 7,026 | 7,135 | 7,185 | 7,170 | 7,098 |  |
|  | Person-years | 108,618 | 112,791 | 113,862 | 113,521 | 111,188 |  |  | 132,213 | 139,704 | 144,187 | 147,115 | 147,680 |  |
|  | Cases | 368 | 386 | 356 | 416 | 390 |  |  | 206 | 222 | 201 | 194 | 218 |  |
|  | ASR^1)^ | 331 | 336 | 308 | 364 | 350 |  |  | 151 | 155 | 138 | 132 | 151 |  |
|  | HR2 (95% CI)^2,3)^ | 1.0 (ref) | 1.17 (1.00-1.36) | 1.12 (0.95-1.32) | **1.37 (1.16-1.61)** | **1.30 (1.08-1.55)** | ***<0.01*** |  | 1.0 (ref) | 1.13 (0.92-1.39) | 0.98 (0.79-1.22) | 0.92 (0.73-1.15) | 1.04 (0.82-1.32) | *0.73* |
| CVD | Cases | 354 | 327 | 376 | 391 | 453 |  |  | 212 | 238 | 264 | 300 | 317 |  |
|  | ASR^1)^ | 366 | 322 | 348 | 348 | 375 |  |  | 165 | 178 | 193 | 207 | 200 |  |
|  | HR2 (95% CI)^2,3)^ | 1.0 (ref) | 0.92 (0.78-1.08) | 1.06 (0.90-1.24) | 1.08 (0.91-1.28) | 1.08 (0.90-1.29) | *0.17* |  | 1.0 (ref) | 1.07 (0.87-1.31) | 1.08 (0.88-1.33) | 1.21 (0.98-1.48) | 1.05 (0.84-1.30) | *0.63* |
| Cerebrovascular | Cases | 135 | 120 | 149 | 150 | 174 |  |  | 76 | 90 | 106 | 125 | 146 |  |
| disease | ASR^1)^ | 134 | 116 | 138 | 133 | 144 |  |  | 57 | 66 | 77 | 86 | 91 |  |
|  | HR2 (95% CI)^2,3)^ | 1.0 (ref) | 0.87 (0.67-1.14) | 1.13 (0.87-1.47) | 1.15 (0.88-1.51) | 1.17 (0.88-1.56) | *0.11* |  | 1.0 (ref) | 1.24 (0.88-1.73) | 1.32 (0.94-1.84) | **1.54 (1.09-2.15)** | **1.58 (1.11-2.25)** | ***<0.01*** |
| **Sodium-to-potassium ratio** | |  |  |  |  |  |  |  |  |  |  |  |  |  |
|  | No. of subjects | 6,062 | 6,188 | 6,248 | 6,278 | 6,272 |  |  | 7,268 | 7,545 | 7,532 | 7,632 | 7,635 |  |
|  | Person-years | 110,837 | 115,017 | 117,513 | 119,331 | 117,961 |  |  | 135,999 | 146,632 | 149,565 | 154,793 | 155,888 |  |
| All-cause death | Cases | 1,379 | 1,441 | 1,560 | 1,628 | 1,875 |  |  | 936 | 946 | 1,029 | 994 | 1,249 |  |
|  | ASR^1)^ | 1,208 | 1,339 | 1,445 | 1,513 | 1,773 |  |  | 674 | 670 | 735 | 707 | 851 |  |
|  | HR2 (95% CI)^2,3)^ | 1.0 (ref) | 1.07 (0.99-1.15) | 1.12 (1.03-1.20) | **1.09 (1.01-1.18)** | **1.23 (1.14-1.33)** | ***<.001*** |  | 1.0 (ref) | 0.94 (0.86-1.04) | 0.99 (0.9-1.09) | 0.92 (0.83-1.01) | 1.02 (0.93-1.12) | *0.71* |
| NCD | Cases | 1,013 | 1,109 | 1,172 | 1,215 | 1,411 |  |  | 659 | 665 | 721 | 705 | 868 |  |
|  | ASR^1)^ | 889 | 1,023 | 1,080 | 1,118 | 1,316 |  |  | 477 | 469 | 509 | 492 | 582 |  |
|  | HR2 (95% CI)^2,3)^ | 1.0 (ref) | 1.11 (1.02-1.21) | 1.12 (1.02-1.22) | **1.09 (0.99-1.19)** | **1.23 (1.12-1.34)** | ***<.001*** |  | 1.0 (ref) | 0.91 (0.82-1.03) | 0.94 (0.84-1.05) | 0.88 (0.79-0.99) | 0.97 (0.86-1.08) | *0.65* |
| Premature NCD^5)^ | No. of subjects | 5,632 | 5,878 | 5,947 | 6,010 | 6,052 |  |  | 6,782 | 7,112 | 7,132 | 7,319 | 7,269 |  |
|  | Person-years | 104,928 | 110,933 | 113,318 | 115,711 | 115,089 |  |  | 128,297 | 139,545 | 143,147 | 149,775 | 150,135 |  |
|  | Cases | 297 | 356 | 339 | 419 | 505 |  |  | 180 | 205 | 211 | 218 | 227 |  |
|  | ASR^1)^ | 285 | 318 | 296 | 355 | 432 |  |  | 140 | 146 | 146 | 144 | 151 |  |
|  | HR2 (95% CI)^2,3)^ | 1.0 (ref) | 1.12 (0.96-1.31) | 1.02 (0.86-1.19) | 1.16 (0.99-1.36) | **1.30 (1.11-1.53)** | ***<.001*** |  | 1.0 (ref) | 1.01 (0.82-1.25) | 0.95 (0.77-1.18) | 0.93 (0.76-1.15) | 0.90 (0.73-1.12) | *0.24* |
| CVD | Cases | 323 | 308 | 389 | 390 | 491 |  |  | 234 | 216 | 253 | 266 | 362 |  |
|  | ASR^1)^ | 283 | 285 | 364 | 369 | 466 |  |  | 168 | 155 | 183 | 189 | 246 |  |
|  | HR2 (95% CI)^2,3)^ | 1.0 (ref) | **0.96 (0.82-1.13)** | **1.13 (0.97-1.32)** | **1.06 (0.91-1.24)** | **1.24 (1.06-1.45)** | ***<0.01*** |  | 1.0 (ref) | 0.84 (0.69-1.02) | 0.96 (0.79-1.16) | 0.97 (0.80-1.17) | 1.14 (0.94-1.37) | ***0.03*** |
| Cerebrovascular | Cases | 110 | 121 | 139 | 152 | 206 |  |  | 87 | 80 | 104 | 121 | 151 |  |
| disease | ASR^1)^ | 96 | 111 | 128 | 149 | 191 |  |  | 62 | 57 | 75 | 89 | 99 |  |
|  | HR2 (95% CI) | 1.0 (ref) | 1.07 (0.82-1.40) | 1.18 (0.91-1.53) | 1.17 (0.90-1.51) | **1.42 (1.10-1.83)** | ***<0.01*** |  | 1.0 (ref) | 0.83 (0.59-1.16) | 1.12 (0.81-1.53) | 1.26 (0.92-1.71) | **1.39 (1.03-1.89)** | ***<0.01*** |

1) ASR, age standardized mortality rate (per 100,000 person-years) 2) HR, hazard ratio; CI, confidence interval. 3) HR2: Adjusted for age (continuous), public health center area, body mass index in kg/m^2^ (<19, 19–22.9, 23–24.9, 25–26.9, and ≥27), physical activity in metabolic equivalent task-hours/day (<30, 30–34.9, 35–39.9, and ≥40), smoking status (never, past, current <20, and current ≥20 cigarette/d), alcohol consumption (none, occasional, 1–149, 150–299, 300–449, and ≥450 g ethanol/week), marital status (married: yes/no), living alone (yes/no), quintile of energy intake, coffee, green tea, SFA, n3PUFA, and potassium (for the analysis of sodium intake), screening examination (gastric photofluorography, gastrointestinal endoscopy, fecal occult blood test, barium enema, colonoscopy), and medication use (hypertension). 4) Linear trends across quintiles of sodium intake or sodium-to-potassium ratio were tested using the median consumption for each quintile as an ordinal variable. 5) For analysis of premature NCD death, subjects aged ≥70 y at baseline were excluded and data for those aged ≥70 y at death were treated as censored observations.

Supplemental TABLE 2. Hazard ratios and 95% confidence intervals for all-cause and cause-specific mortality by quintile of sodium consumption and sodium-to-potassium ratio for men and women combined: the JPHC Study, 1995 and 1998–2018

|  |  | **Men and women combined** (n=83,048) | | | | | *trend p*^4)^ |
| --- | --- | --- | --- | --- | --- | --- | --- |
|  |  | **Q1** | **Q2** | **Q3** | **Q4** | **Q5** |  |
| **Sodium** |  |  |  |  |  |  |  |
|  | No. of subjects | 16,609 | 16,610 | 16,610 | 16,610 | 16,609 |  |
|  | Person-years | 302,392 | 312,554 | 318,640 | 325,953 | 328,362 |  |
| All-cause death | Cases | 3,550 | 3,374 | 3,494 | 3,470 | 3,839 |  |
|  | ASR^1)^ | 1,310 | 1,176 | 1,187 | 1,114 | 1,164 |  |
|  | HR2 (95% CI)^2,3)^ | 1.0 (ref) | 0.98 (0.93-1.03) | 1.02 (0.97-1.08) | 0.99 (0.94-1.05) | 1.05 (0.99-1.12) | ***0.06*** |
| NCD | Cases | 2,517 | 2,467 | 2,596 | 2,535 | 2,745 |  |
|  | ASR^1)^ | 924 | 852 | 875 | 806 | 826 |  |
|  | HR2 (95% CI)^2,3)^ | 1.0 (ref) | 1.00 (0.95-1.07) | 1.06 (.998-1.13) | 1.02 (0.95-1.09) | 1.06 (0.99-1.13) | *0.12* |
| Premature NCD^5)^ | No. of subjects | 15440 | 15501 | 15552 | 15538 | 15436 |  |
|  | Person-years | 285,752 | 296,123 | 302,957 | 309,657 | 310,672 |  |
|  | Cases | 808 | 709 | 739 | 640 | 659 |  |
|  | ASR^1)^ | 275 | 236 | 240 | 205 | 214 |  |
|  | HR2 (95% CI)^2,3)^ | 1.0 (ref) | 1.02 (0.92-1.14) | 1.14 (1.02-1.27) | 1.06 (0.94-1.20) | **1.16 (1.02-1.33)** | ***0.03*** |
| Cancer | Cases | 1,350 | 1,389 | 1,420 | 1,348 | 1,414 |  |
|  | ASR^1)^ | 486 | 473 | 468 | 422 | 424 |  |
|  | HR2 (95% CI)^2,3)^ | 1.0 (ref) | 1.07 (0.99-1.16) | 1.12 (1.03-1.21) | 1.04 (0.95-1.14) | 1.08 (0.98-1.19) | *0.30* |
| CVD | Cases | 937 | 847 | 941 | 953 | 1,067 |  |
|  | ASR^1)^ | 351 | 297 | 324 | 306 | 322 |  |
|  | HR2 (95% CI)^2,3)^ | 1.0 (ref) | 0.92 (0.83-1.02) | 1.01 (0.91-1.12) | 1.00 (0.90-1.11) | 1.03 (0.92-1.15) | *0.26* |
| Heart disease | Cases | 507 | 461 | 454 | 499 | 517 |  |
|  | ASR^1)^ | 191 | 163 | 157 | 162 | 156 |  |
|  | HR2 (95% CI)^2,3)^ | 1.0 (ref) | 0.94 (0.82-1.07) | 0.90 (0.78-1.04) | 0.98 (0.84-1.13) | 0.93 (0.79-1.09) | *0.60* |
| Cerebrovascular | Cases | 323 | 316 | 387 | 357 | 463 |  |
| disease | ASR^1)^ | 119 | 110 | 132 | 113 | 140 |  |
|  | HR2 (95% CI)^2,3)^ | 1.0 (ref) | 1.00 (0.85-1.19) | 1.26 (1.06-1.48) | 1.13 (0.94-1.35) | **1.36 (1.13-1.63)** | ***<.001*** |
|  |  |  |  |  |  |  |  |
| **Sodium-to-potassium ratio** | |  |  |  |  |  |  |
|  | No. of subjects | 16,609 | 16,610 | 16,610 | 16,610 | 16,609 |  |
|  | Person-years | 307,104 | 315,593 | 320,277 | 323,374 | 321,553 |  |
| All-cause death | Cases | 3,176 | 3,203 | 3,439 | 3,598 | 4,311 |  |
|  | ASR^1)^ | 1,003 | 1,059 | 1,167 | 1,229 | 1,481 |  |
|  | HR2 (95% CI)^2,3)^ | 1.0 (ref) | 0.99 (0.94-1.05) | 1.01 (0.96-1.06) | 0.99 (0.94-1.04) | **1.09 (1.03-1.14)** | ***<.001*** |
| NCD | Cases | 2,205 | 2,334 | 2,554 | 2,615 | 3,152 |  |
|  | ASR^1)^ | 698 | 769 | 858 | 880 | 1,067 |  |
|  | HR2 (95% CI)^2,3)^ | 1.0 (ref) | 1.02 (0.96-1.08) | 1.04 (0.98-1.11) | 1.00 (0.94-1.06) | **1.09 (1.03-1.16)** | ***0.01*** |
| Premature NCD^5)^ | No. of subjects | 15107 | 15373 | 15573 | 15681 | 15733 |  |
|  | Person-years | 284,591 | 297,141 | 304,871 | 309,551 | 309,006 |  |
|  | Cases | 548 | 604 | 691 | 769 | 943 |  |
|  | ASR^1)^ | 194 | 202 | 223 | 244 | 300 |  |
|  | HR2 (95% CI)^2,3)^ | 1.0 (ref) | 0.98 (0.87-1.10) | 1.01 (0.90-1.14) | 1.05 (0.93-1.18) | 1.10 (0.98-1.24) | ***0.03*** |
| Cancer | Cases | 1,174 | 1,312 | 1,394 | 1,394 | 1,647 |  |
|  | ASR^1)^ | 374 | 428 | 459 | 455 | 544 |  |
|  | HR2 (95% CI)^2,3)^ | 1.0 (ref) | 1.06 (0.97-1.15) | 1.04 (0.95-1.13) | 0.97 (0.89-1.06) | 1.05 (0.97-1.14) | *0.76* |
| CVD | Cases | 841 | 807 | 898 | 992 | 1,207 |  |
|  | ASR^1)^ | 265 | 269 | 308 | 344 | 417 |  |
|  | HR2 (95% CI)^2,3)^ | 1.0 (ref) | 0.95 (0.85-1.05) | 1.00 (0.90-1.10) | 1.01 (0.91-1.12) | **1.11 (1.01-1.23)** | ***<0.01*** |
| Heart disease | Cases | 448 | 418 | 454 | 508 | 610 |  |
|  | ASR^1)^ | 141 | 140 | 157 | 176 | 213 |  |
|  | HR2 (95% CI)^2,3)^ | 1.0 (ref) | 0.93 (0.81-1.07) | 0.97 (0.84-1.11) | 0.99 (0.86-1.13) | 1.08 (0.94-1.24) | *0.13* |
| Cerebrovascular | Cases | 299 | 310 | 348 | 393 | 496 |  |
|  | ASR^1)^ | 94 | 103 | 118 | 136 | 169 |  |
| disease | HR2 (95% CI)^2,3)^ | 1.0 (ref) | 1.02 (0.86-1.21) | 1.09 (0.92-1.29) | 1.11 (0.94-1.30) | **1.27 (1.08-1.50)** | ***<0.01*** |

1) ASR, age standardized mortality rate (per 100,000 person-years) 2) HR, hazard ratio; CI, confidence interval. 3) HR2: Adjusted for sex, age (continuous), public health center area, body mass index in kg/m^2^ (<19, 19–22.9, 23–24.9, 25–26.9, and ≥27), physical activity in metabolic equivalent task-hours/day (<30, 30–34.9, 35–39.9, and ≥40), smoking status (never, past, current <20, and current ≥20 cigarette/d), alcohol consumption (none, occasional, 1–149, 150–299, 300–449, and ≥450 g ethanol/week), marital status (married: yes/no), living alone (yes/no), quintile of energy intake, coffee, green tea, SFA, n3PUFA, and potassium (for the analysis of sodium intake), screening examination (gastric photofluorography, gastrointestinal endoscopy, fecal occult blood test, barium enema, colonoscopy), and medication use (hypertension). 4) Linear trends across quintiles of sodium intake or sodium-to-potassium ratio were tested using the median consumption for each quintile as an ordinal variable. 5) For analysis of premature NCD death, subjects aged ≥70 y at baseline were excluded and data for those aged ≥70 y at death were treated as censored observations.
